# Modification of the nuclear pleomorphism score in the Modified Bloom-Richardson grading for invasive breast cancer

**DOI:** 10.1371/journal.pone.0327860

**Published:** 2025-07-18

**Authors:** Azam Hilmi Mohd Zain, Seoparjoo Azmel Mohd Isa, Nur Asyilla Che Jalil, Suhaily Mohd Hairon, Syed Sana Abrar

**Affiliations:** 1 Department of Pathology, Hospital Umum Sarawak, Jalan Hospital, Kuching, Sarawak, Malaysia; 2 Department of Pathology, School of Medical Sciences, Universiti Sains Malaysia, Kelantan, Malaysia; 3 Department of Community Medicine, School of Medical Sciences, Universiti Sains Malaysia, Kelantan, Malaysia; University of Arkansas for Medical Sciences, UNITED STATES OF AMERICA

## Abstract

**Objective:**

To improve prognostic assessments in breast cancer by evaluating the efficacy of the modified Nottingham grading system, specifically focusing on nuclear pleomorphism through measurements of largest nuclear size (LNS) and nuclear size difference (NSD).

**Methods:**

This cross-sectional study involved 140 invasive breast cancer cases at Hospital Universiti Sains Malaysia. The study consisted of two phases: Phase 1, which developed nuclear scoring criteria using histopathological images from 2013–2017, and Phase 2, which validated these criteria on cases from 2018–2019. Two sets of samples were included for the study, with 120 cases analyzed in Phase 1 and 53 cases in Phase 2. Descriptive statistics and normality tests assessed differences in LNS and NSD across original nuclear grades.

**Results:**

Significant differences were found in LNS and NSD across original nuclear grades (p < 0.05), with fair to moderate agreement between the modified grading system and pathologist assessments (Kappa = 0.367).

**Conclusion:**

Incorporating LNS and NSD into the assessment of nuclear pleomorphism shows potential to improve objectivity in breast cancer grading. However, further validation using larger datasets and automated image analysis is necessary to refine this approach and assess its clinical applicability..

## Introduction

Breast cancer is the most frequently diagnosed cancer among women worldwide, accounting for approximately 25.1% of cases [1]. Accurate histological grading is essential for developing effective treatment strategies and predicting patient outcomes. Tumor grade significantly influences prognosis, with lower-grade tumors often associated with better survival outcomes, while higher-grade tumors are linked to early recurrence and metastasis [2].

The prognostic implications of tumor grading are well-documented. A study demonstrated significant differences in survival rates between patients with grade I tumors and those with grades II and III tumors (p < 0.0001) [3]. High-grade tumors, characterized by poor differentiation, exhibit a greater likelihood of systemic recurrences and mortality associated with metastasis [4]. Tumor grade is a crucial component of the Nottingham Prognostic Index (NPI), an important prognostic tool in breast cancer management. The NPI incorporates lymph node involvement, tumor size, and tumor grade to create a formula that predicts 5-year survival following surgery for breast cancer [5,6]. Notably, among the three features used in the Nottingham index, tumor grade emerged as the most indicative and significant prognostic factor [7].

The Nottingham modification of the Bloom-Richardson system, which enhances the reproducibility of the original grading criteria, has received endorsement from several authoritative organizations, including the College of American Pathologists, the World Health Organization, the Commission on Cancer (CoC), and the National Accreditation Program for Breast Centres (NAPBC) [8]. This system evaluates tumor differentiation based on three morphological features: (a) the percentage of tubule or gland formation, (b) the degree of nuclear pleomorphism, and (c) the mitotic count.

A common criticism of the grading system is its reproducibility [9]. Studies assessing grading reproducibility have shown modest-to-good levels of agreement between independent observers, with mostly fair-to-moderate agreement observed for its three components [10,11]. Among these, nuclear pleomorphism is the least reproducible parameter [12]. Although efforts have been made to reinforce adherence to grading criteria, Meyer (2005) noted that these initiatives did not significantly improve inter-observer agreement [11]. The absence of precise standards or formal measurements for objectively classifying nuclear size and shape further compounds this issue. This inherent subjectivity in the grading system limits its reproducibility and agreement, raising concerns about its impact on patient prognosis and decision-making [11].

Numerous efforts to standardize histological grading have focused on improving objectivity, such as refining mitotic figure counting techniques [13]. However, assessing nuclear pleomorphism remains challenging due to its subjective nature. Recent advancements in digital histopathology have enabled more objective analyses of nuclear features, contributing to a more standardized grading process [14]. The cross-sectional area of nuclei, typically oval to circular in shape, can be characterized by parameters such as surface area, perimeter, and diameter in two-dimensional images [15]. Despite the well-established clinical relevance of grading, the predictive value of the Nottingham system is hindered by its subjective components, particularly nuclear pleomorphism, which lacks standardized evaluation criteria.

This study aims to explore the utility of nuclear diameter in grading pleomorphism within the framework of the Modified Bloom-Richardson system for invasive breast carcinoma. We propose a modified scoring system for nuclear pleomorphism that incorporates quantifiable nuclear features and evaluate its validity and reliability in comparison to traditional method

## Methodology

### Study design

A cross-sectional, two-phase study was conducted at Hospital Universiti Sains Malaysia from 2018 to 2019. In Phase 1, we analyzed potential parameters and modified the nuclear scoring criteria using histopathology images of invasive breast cancer diagnosed between 2013 and 2017. Phase 2 involved validating these modified criteria with histopathology images of invasive breast cancer diagnosed from 2018 to 2019. Ethical approval for this study was granted by the Human Ethical Committee of Universiti Sains Malaysia.

### Study population and sample size determination

Two sets of samples were utilized for this study. For Phase 1,140 cases of invasive breast cancer were selected from 160 cases registered in the HUSM online pathology system between 2013 and 2017. The sample size was determined based on recommendations by Huma and Waheed (2013), [16] who proposed that 120 cases are adequate for establishing reference ranges in pathology-based studies. To accommodate potential exclusions, an additional 20% was added. After excluding cases with inadequate material or incomplete data, a final total of 120 cases was included for analysis. For validation (Phase 2), 53 invasive breast cancer cases were selected from 60 registered cases diagnosed between 2018 and 2019. The ideal sample size for this phase was calculated to be 49 using a kappa table, with a minimum kappa limit of 0.5 and an expected kappa of 0.8 [17]. Inclusion criteria comprised invasive breast cancer cases with available H&E slides, while exclusion criteria included metastatic cases and those with inadequate histopathology reports.

### Data collection

Cases were reviewed consecutively through the computerized registry system (Laboratory Information System, LIS) in the Pathology Department at HUSM. All cases diagnosed as “invasive breast cancer” or “invasive ductal carcinoma” between 2013 and 2017 were manually identified. The corresponding formal pathology reports were examined to confirm eligibility. Histological slides were retrieved following standard HUSM laboratory protocols, and tumor sections were reviewed microscopically to confirm the diagnosis and assess slide adequacy. Inclusion criteria consisted of histologically confirmed invasive breast cancer cases with available and adequate hematoxylin and eosin (H&E) stained slides. Exclusion criteria included metastatic disease, incomplete pathology reports, or inadequate tissue quality. For cases with faded staining due to aging, re-sectioning and re-staining with H&E were performed to ensure diagnostic quality. This consecutive case selection process was designed to minimize selection bias and ensure representativeness of the sample.

### Measurement of nuclear sizes

All slides were microscopically reviewed to identify the most pleomorphic tumor areas, considering tumor heterogeneity. An Olympus BX51 microscope, equipped with a 10x/22 mm ocular lens and Olympus DP72 camera, was utilized at 40x magnification to capture high-power fields containing tumor cells as JPG images (1360x1024 pixels), labeled according to their laboratory numbers for reference.

**Fig 1:**
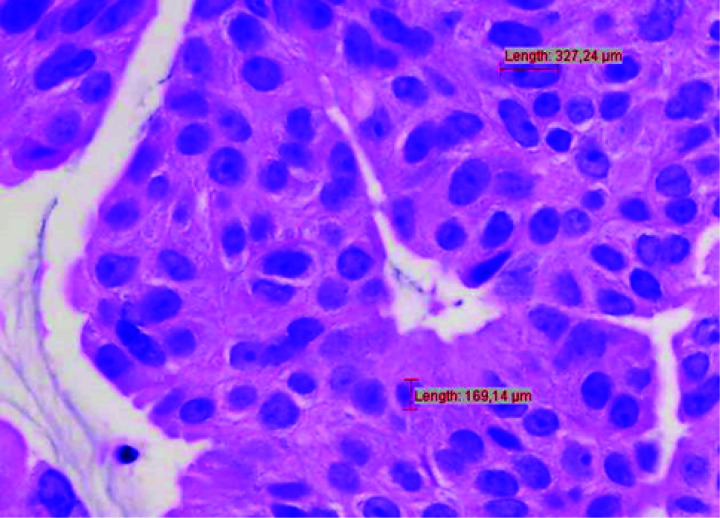
Measurement of nuclear diameter, 400x H&E.

Image analysis was performed using Cell^F software. The overall nuclear sizes were recorded, focusing on the largest tumor cells in each image, measured manually along the cell’s longest axis. This process involved placing the cursor on one pole of the nuclear membrane, dragging it to the opposite pole, and recording the length as the “largest nuclear size” (LSN). A similar method was applied for the smallest tumor cells, termed “smallest nuclear size” (SNS). An example of this measurement is shown in Fig 1.

Measurements were conducted by a single researcher using consistent settings for the microscope, camera, and software. The values were standardized with one pixel representing 6.5 µm. While measurements were conducted by a single trained researcher using standardized equipment, magnification, and software settings, we acknowledge that manual selection of nuclei may introduce some measurement variability. To mitigate this only tumor cells with complete, well-defined nuclear membranes were included; crushed or obscured cells, as well as multinucleated giant cells, were excluded. In cases where the largest or smallest cells were challenging to identify, multiple measurements from several selected cells were averaged for precision. The nuclear size difference (NSD) was calculated by subtracting SNS from LSN, documenting LSN, SNS, and NSD for each slide.

### Statistical analysis

Statistical analysis was conducted using SPSS version 25.0. Statistical analysis included: (1) testing potential variables, (2) determining cut-off values for the variables, (3) modifying nuclear grading based on these variables, and (4) validating the new model. LSN, SNS, and NSD were the three variables used in our statistical analysis. Mean and standard deviation for each variable were calculated, and normality testing was performed to ascertain whether the data followed a normal distribution using Shapiro-Wilk test. For variables meeting this criterion, analysis of variance (ANOVA) with Bonferroni correction was conducted to determine significant differences in means across the three nuclear scores. Variables demonstrating significant mean differences were incorporated into the construction of the new model.

Once the variables were identified, cut-off points for each were established to represent the three nuclear scores: score 1 (mild), score 2 (moderate), and score 3 (marked pleomorphism). This classification was based on the standard deviation for each variable. After determining the ranges, each variable was assigned the corresponding nuclear score and tested against the original scores to assess agreement. The model yielding the best results was chosen as the Modified Grading System.

The Modified Grading System was then validated by applying it to a separate set of breast cancer images. Agreement was assessed by comparing the nuclear scores generated using the modified technique with the original scores assigned by pathologists. To evaluate the level of concordance between the two scoring methods, concordant and discordant pair analysis, Cohen’s kappa statistic, and Fisher’s exact test were performed. Cohen’s kappa was used to quantify inter-scale agreement, with values ≥0.6 interpreted as indicating good reliability between the modified and original nuclear scores

## Results

Based on the histopathology reports, the majority of cases exhibited moderate nuclear pleomorphism (80 cases, or 66.6%), while cases with mild pleomorphism were the least common (13 cases, or 10.8%). The distribution of cases showed a ratio of 1:6:2 for mild, moderate, and marked pleomorphism.

### Analysis of nuclear sizes

Descriptive statistics indicated that the largest nuclear size (LNS) of breast cancer cells was 710.38 µm, which is approximately seven times larger than the smallest nuclear size (111.26 µm). The widest gap observed between nuclear sizes in one case was 530.14 µm (Table 1). Normal distribution was observed through histogram and box and whisker plot for SNS, LNS, and NSD.

**Table 1:** Mean, Standard Deviation, Kolmogorov-Smirnov^a^ and Shapiro-Wilk Normality Test of Measured Variables (n = 120).

Variable	Lowest Value (µm)	Highest Value (µm)	Mean (sd) (µm)	Kolmogorov-Smirnov^a^			Shapiro-Wilk		
				Statistic	df	Significance	Statistic	df	Significance
**Smallest Nuclear Size (SNS)**	111.26	291.33	184.73 (36.30)	.050	120	.200^*^	.989	120	.438
**Largest Nuclear Size (LNS)**	181.24	710.38	340.49 (91.10)	.107	120	.002	.943	120	.000
**Nuclear Size Difference (NSD)**	26.72	530.14	155.76 (78.56)	.101	120	.004	.880	120	.000

*a.Lilliefors Significance Correction.*

### Analysis of variance (ANOVA)

No significant differences were noted in the smallest nuclear size (SNS) across the original nuclear scores (1, 2, and 3) (Table 2). However, significant differences were observed in LNS, as revealed by post hoc analysis using Bonferroni’s procedure. Specifically, LNS was significantly larger in Grade 3 compared to Grade 1 (p < 0.001) and Grade 2 (p = 0.001), indicating a marked increase in nuclear size as pleomorphism intensified. Comparatively, the difference between Grade 1 and Grade 2 approached significance, with a p-value of 0.080.

**Table 2:** Mean of SNS and LNS between Original Nuclear Grade (n = 120).

Original Nuclear Grade	N	Smallest Nuclear Size			Largest Nuclear Size		
		Mean (sd)	F-statistics (df)	p-value	Mean (sd)	F-statistics (df)	p-value
1	13	180.21 (31.33)	1.945 (2,119)	0.148	247.37 (67.34)	11.698 (2,119)	<0.001
2	80	181.41 (34.82)			330.69 (85.81)		
3	27	196.74 (36.30)			401.36		

### Nuclear Size Difference

Post hoc analysis for the nuclear size difference (NSD) also demonstrated significant results. The differences in LNS among the original nuclear grades (1, 2, and 3) were statistically significant, with Grade 1 showing a smaller LNS compared to both Grade 2 (p = 0.037) and Grade 3 (p < 0.001). These findings underscore significant disparities in nuclear size associated with increasing pleomorphism (Table 3).

**Table 3:** Mean of NSD between Original Nuclear Grade.

Original Nuclear Grade	N	Mean (sd)	F-statistics (df)	p-value
1	13	94.16 (54.18)	11.101 (2,119)	<0.001
2	80	149.28 (70.72)		
3	27	204.61 (84.81)		

### New cut-off values and distribution of cases

The new cut-off values for LNS and NSD were derived from the standard deviation of Grade 2 cases. Applying these cut-offs led to a reassignment of nuclear scores for all 120 cases, revealing a shift in the distribution of Grade 3 cases. Specifically, Grade 3 cases decreased from 27 to 22 using LNS, and further to 17 with NSD-based scoring, accompanied by an increase in Grades 1 and 2 (Table 4).

**Table 4:** Distribution of Cases by Nuclear Grade: New Variables VS Original Grading.

	Based on Largest Nuclear Size, n(%)	Based on Nuclear Size Difference, n(%)	Based on Original Grading, n(%)
**Grade 1**	16 (13.3)	14 (11.7)	13
**Grade 2**	82 (68.3)	89 (74.2)	80
**Grade 3**	22 (18.3)	17(14.2)	27

### Testing agreement for new grades

The agreement between the new grading system and the original nuclear scores was evaluated using kappa statistics. For the LNS-based grading system, a kappa value of 0.250 indicated fair agreement (p < 0.001), with a concordance rate of 63.3% and a discordance rate of 36.7%. In contrast, the NSD-based grading showed a lower kappa value of 0.150, signifying only slight agreement (p = 0.034), with 60.8% of cases in concordance and 39.2% in discordance. When LNS and NSD were combined to form a modified nuclear grading system, the concordance rate increased to 65.0%, with a corresponding discordance rate of 35.0%, reflecting fair agreement (Kappa = 0.256, p < 0.001).

### Validation of the modified nuclear grading system

For LNS, 60.4% of 53 cases were concordant with the original nuclear grade, while 39.6% were discordant, particularly between borderline grades (1 vs. 2 and 2 vs. 3). The Kappa value was 0.235 (p = 0.058), indicating fair agreement. For NSD, concordance was higher at 69.8%, with 30.2% discordant cases, including one critical discordance between Grade 1 and Grade 3. The Kappa value was 0.453 (p < 0.001), indicating moderate agreement.

Using the combined grading system (LNS + NSD), concordance was observed at 66.1%, with 33.9% discordant. Most discrepancies occurred between adjacent grades, with one critical discordance noted between Grade 1 and Grade 3. The Kappa value was 0.367 (p = 0.002), indicating moderate agreement (Table 5).

**Table 5:** Concordant and Discordant Pair Analysis for Modified Nuclear Grading System (n = 53).

		Original Nuclear Score			Total
1	2	3	
**Modified Nuclear Grade (Combination of LNS and NSD)**	1	0	3	0	3
	2	3	22	5	30
	3	1	6	13	20
**Total**		4	31	18	53

*Kappa value = 0.367, p-value=0.002.*

In summary, the modified grading system demonstrates fair to moderate agreement with the original grades assigned by pathologists, particularly effective at distinguishing between adjacent grades, suggesting its potential utility for clinical application.

## Discussion

Breast cancer remains the most prevalent cancer among women and the second leading cause of cancer-related mortality worldwide [1]. An accurate histopathological diagnosis, supported by grading systems and hormonal evaluations, is crucial for informing treatment strategies and prognostic assessments [8]. Histological grading has significant prognostic implications, as high-grade tumors are associated with poorer outcomes compared to low-grade tumors [2]. Despite the introduction of new prognostic markers, the evaluation of tumor grade continues to be essential [18]. Traditional grading systems, established by Greenough and later refined by Bloom and Richardson, focus on three key parameters: tubular formation, mitotic figures, and nuclear pleomorphism.

To enhance objectivity and inter-observer reliability, ongoing modifications to the grading process have been implemented. Mitosis is now assessed using defined cutoff points, and tubular formation is quantified per grade. However, the evaluation of nuclear pleomorphism remains ambiguous. The WHO guidelines for classifying nuclear pleomorphism rely on relative comparisons of nuclear sizes, leading to variability in grading due to subjective interpretations by pathologists. Research has indicated that nuclear pleomorphism is a significant weakness in the Modified Bloom-Richardson grading system, affecting agreement and reproducibility [11].

This study introduces a novel approach that prioritizes nuclear diameter as a key indicator for nuclear scoring, recognizing nucleomegaly as a hallmark of malignancy [19,20]. The decision to utilize nuclear diameter is based on its relative ease of measurement and stability across varying degrees of pleomorphism. Previous research has explored different metrics related to nuclear size, such as nuclear perimeter and area, with varying complexities [16,21,22]. This study contributes a unique methodology, as no similar studies have been reported.

Our modified classification system is aligned with a machine learning framework, incorporating a “training” phase (Phase 1) utilizing data from 120 breast cancer cases and a “testing” phase (Phase 2) involving 53 cases for validation. This structure emphasizes that extensive training enhances system design, while thorough testing ensures reliability [14].

In analyzing data from the 120 cases diagnosed between 2013 and 2017, the distribution of tumor grades was consistent with previous literature, revealing Grade 2 tumors as the most prevalent. Significant differences in mean values for the largest nuclear size (LNS) and nuclear size difference (NSD) were noted across the three original nuclear scores (p < 0.001), while no significant difference was observed for the smallest nuclear size (SNS) (p = 0.148). These findings suggest that LNS and NSD effectively represent the three grades of nuclear pleomorphism. The new scoring model is based on carefully determined cutoff values for LNS and NSD, with both metrics demonstrating enhanced agreement when analyzed collectively (Kappa = 0.256).

These findings are not only statistically significant but also make sense biologically. The largest nuclear size (LNS) reflects nucleomegaly, a common feature in cancer cells that indicates increased DNA content and cellular atypia. The nuclear size difference (NSD), on the other hand, captures how much nuclear size varies within the tumor, a sign of cellular instability and poor differentiation. Together, these features serve as meaningful indicators of nuclear pleomorphism, which is a key marker of tumor aggressiveness and malignancy.

Validation on the 53 cases yielded moderate agreement (Kappa = 0.367), consistent with Kappa values reported among pathologists [11]. The concordant-discordant pair analysis revealed a 66.1% concordance and 33.9% discordance, primarily between intermediate scores. Notably, a critical discordance was identified when a case rated Grade 1 by the original score was assigned Grade 3 in the modified scoring system. This discrepancy highlights the potential for variability, particularly with NSD reflecting non-uniformity in malignant nuclei.

The observed discordances may arise from measuring LNS and SNS from atypical tumor cell morphologies, leading to inaccurate representations of overall tumor characteristics. For instance, the overestimation of LNS from giant cells and the underestimation of SNS from apoptotic bodies can inflate NSD scores. Despite this, the findings indicate that LNS and NSD can effectively capture varying degrees of nuclear pleomorphism, aligning with the original nuclear scores with fair reliability.

Although not included in the final model, features such as solidity, eccentricity, and orientation entropy were explored during the preliminary analysis phase due to their known biological relevance. Solidity represents nuclear contour irregularities commonly seen in high-grade tumors; eccentricity reflects nuclear elongation, which may indicate infiltrative patterns; and orientation entropy quantifies the randomness of nuclear alignment, potentially corresponding to loss of tissue architecture. While these features showed limited discriminatory value in our dataset, future studies may benefit from incorporating them in conjunction with additional morphological or molecular parameters.

The ability to replicate the original nuclear score is essential for validating our modified system. Given the subjective nature of grading, our method’s reliability was benchmarked against established standards set by experienced pathologists. While our validation utilized a limited sample size, further research with larger datasets is necessary to enhance the evaluation and applicability of this new scoring system. This study provides a foundation for improved grading methods in breast cancer histopathology, contributing to more accurate prognostic assessments and potentially influencing patient management strategies.

### Limitations

Our study on nuclear pleomorphism has several limitations that should be considered. Firstly, manual measurement of nuclear sizes introduces potential variability. Although all measurements were conducted by a single trained researcher using a standardized imaging setup, intra-observer variability may still occur. Unlike fully automated systems that analyze all nuclei in an image, we selected an average of 3 to 4 pleomorphic cells per image. This selective measurement approach may not fully represent the heterogeneity of the tumor and introduces bias in identifying the most pleomorphic nuclei, especially in highly cellular areas. While selective region-of-interest (ROI) analysis has been shown to yield reliable results as demonstrated by Dalle et al. (2009) with 40–50 nuclei per image [23], our limited manual sampling could lead to quantification error.We also acknowledge the limitations of not incorporating automated image analysis. Although we recommend AI-based or machine learning tools in future studies to improve reproducibility and reduce subjectivity, preliminary attempts at using automation during this study were hindered by poor staining quality and inconsistent nuclear segmentation. These technical issues led us to adopt a manual approach in this phase Nevertheless, integrating automated image analysis or machine learning particularly in conjunction with whole slide imaging (WSI) could enhance accuracy and efficiency in routine workflows [14].

The quality of histopathological slides posed another challenge, as many lacked clear cell outlines and proper staining intensity, limiting our sample size. Older H&E slides often exhibited deterioration, affecting their suitability for accurate analysis.

Our study’s image quality was compromised by using lower-resolution JPEG files, hindering the assessment of subtle nuclear details, which are critical for accurate evaluations [16] Additionally, nuclear size variation complicates comparisons to normal breast epithelial cells, for which specific measurements are lacking in the literature [24].

Our validation phase included only 53 cases, which may restrict the statistical power and generalizability of the agreement analysis. A larger, more diverse validation dataset is needed to confirm the performance and reproducibility of the Modified Grading System. Furthermore, the study was conducted at a single institution; Hospital Universiti Sains Malaysia, using a specific microscope-camera-software setup. Therefore, the grading thresholds established in this study are calibrated to this particular imaging context. This may limit the model’s generalizability to other clinical environments or institutions with different equipment and protocols. External validation with multi-institutional datasets is warranted to assess its broader applicability..

Lastly, potential confounding factors such as tumor biology (e.g., molecular subtype, grade), hormonal status, and patient demographic differences were not accounted for in this analysis. These variables can influence nuclear morphology and may impact the accuracy of the grading model. Future studies should consider integrating clinical-pathological data and stratified analyses to reduce residual confounding and improve model robustness.

Despite these limitations, advancements in digital pathology, such as whole slide imaging (WSI), could enhance our methodology. WSI allows for multiresolution imaging, preserving critical details and facilitating better selection and measurement of nuclei [25]. Moreover, integrating automated image analysis with deep learning capabilities can improve reproducibility and mitigate observer variability [14]. Utilizing publicly available datasets, like the BreakHis or “Breast Cancer Histopathological Image Classification” dataset, may also alleviate training limitations and foster more extensive validation studies [26].

## Conclusion

In summary, the measurement of nuclear diameter through LNS and NSD shows promise in capturing varying degrees of nuclear pleomorphism. While the proposed cutoff points may contribute to a more objective grading system, this study represents a preliminary step. Further validation using larger, multicenter datasets and automated image analysis is necessary to confirm the reproducibility and generalizability of the modified grading system. As digital pathology continues to evolve, integrating quantitative methods such as this could support future advancements in histopathological grading.

## Supporting information

S1 Dataset Modification of the Nuclear Pleomorphism. (XLSX)
